# A Reparametrized CNN Model to Distinguish Alzheimer's Disease Applying Multiple Morphological Metrics and Deep Semantic Features From Structural MRI

**DOI:** 10.3389/fnagi.2022.856391

**Published:** 2022-05-26

**Authors:** Zhenpeng Chen, Xiao Mo, Rong Chen, Pujie Feng, Haiyun Li

**Affiliations:** Beijing Key Laboratory of Fundamental Research on Biomechanics in Clinical Application, School of Biomedical Engineering, Capital Medical University, Beijing, China

**Keywords:** reparametrized CNN, Alzheimer's disease, structural MRI, multiple morphological metrics, deep semantic features

## Abstract

It is of potential clinical value to improve the accuracy of Alzheimer's disease (AD) recognition using structural MRI. We proposed a reparametrized convolutional neural network (Re-CNN) to discriminate AD from NC by applying morphological metrics and deep semantic features. The deep semantic features were extracted through Re-CNN on structural MRI. Considering the high redundancy in deep semantic features, we constrained the similarity of the features and retained the most distinguishing features utilizing the reparametrized module. The Re-CNN model was trained in an end-to-end manner on structural MRI from the ADNI dataset and tested on structural MRI from the AIBL dataset. Our proposed model achieves better performance over some existing structural MRI-based AD recognition models. The experimental results show that morphological metrics along with the constrained deep semantic features can relatively improve AD recognition performance. Our code is available at: https://github.com/czp19940707/Re-CNN.

## 1. Introduction

Alzheimer's disease (AD) is an irreversible neurodegenerative disease (Jagust, 2013) arised from a progressive neuron and synapse loss, with the resulting brain tissue atrophy. As seen from the pathological and clinical manifestations of AD, significant atrophy can be observed in early disease stages in the hippocampus and entorhinal cortex (Pennanen et al., 2004). The brain tissue atrophy may be visible on high-resolution structural MRI, and structural MRI measures can discriminate AD from healthy control (NC). Schmitter et al. (2015) utilized the voxel-based morphometry (VBM) to extract the hippocampus volume to discriminate AD from NC and achieved an accuracy of 83%. Koikkalainen et al. (2011) used deformation-based morphometry (DBM) to obtain structural MRI features and reached an accuracy of 86%. Park et al. (2012) computed cortical thickness (CTH) and sulcus depth (SD) with surface-based morphometry (SBM) and attained an accuracy of 85%. Ma et al. (2020) integrated gray matter volume (GMV), Jacobian determinant (JDV), CTH, SD, gyrification index (GI), and fractal dimension (FD) to discriminate mild cognitive impairment (MCI) from NC with random forest and achieved an accuracy of 80%. Long et al. (2018) proposed a comparative atlas-based recognition to recognize MCI from NC, GMV, white matter volume (WMV), and cerebrospinal fluid volume (CRFV) were calculated and reported accuracies of 83, 92, and 89% with AAL-90, BN-246, and AAL-1024 atlas, respectively. These studies needed to determine the region of interest (ROI) for computing morphological metrics, and ROIs often included voxel-level and region-level. It is obvious that neither voxel-level nor region-level based approaches can cover whole brain pathological regions. Deep semantic features may play an important role in AD recognition. Convolutional neural networks (CNNs) were considered backbone structures. Aderghal et al. (2018) designed deep CNNs to structural MRI using transfer learning adopted 188 AD and 228 NC subjects and achieved an accuracy of 90%. Pan et al. (2020) proposed a novel model for structural MRI combining CNN and ensemble learning for AD recognition, collected 137 AD and 162 NC subjects, and reached an accuracy of 84%. Qiu et al. (2020) proposed an interpretable deep learning framework to structural MRI for AD recognition, chose 188 AD subjects and 229 NC subjects, and attained an accuracy of 83%. Lian et al. (2018) proposed a hierarchical fully convolutional network, employed 358 AD subjects and 429 NC subjects, and achieved an accuracy of 90%. CNN based models apply a large number of convolutional kernels for feature extraction, the produced features are often redundant and correlated. An adaptive global pooling layer was used to filter the features (He et al., 2016; Huang et al., 2017; Tan and Le, 2019), and replaced the feature maps with their mean values to achieve dimensionality reduction. However, after the pooling layer, the produced feature's dimensionality is still high. The accuracy of structural MRI based AD recognition is hard to be improved (both morphological based and CNN based) due to the structural similarity between samples and limited training data. How to filter the redundant information effectively and extract the most distinguishing features from structural MRI scans is a challenge.

To improve the accuracy of recognition of structural MRI in AD, we built an end-to-end deep learning model Re-CNN on structural MRI for AD recognition. The main innovations of this article are as follows: (1) combined deep semantic features with morphological metrics (derived from VBM) for AD recognition. (2) created a reparametrized module to impose similarity constraints on deep semantic features, retained the most distinguishing features, reduce feature dimensionality, and improve AD recognition performance.

## 2. Method

In this article, a reparametrized convolutional neural network (Re-CNN) model was proposed on structural MRI to identify AD by applying morphological metrics and deep semantic features. The reparametrized module was designed to constrain the similarity of deep semantic features, achieving the most distinguishing features. The morphological metrics were derived from VBM (Jing et al., 2018; Zhao et al., 2021), where GMV, white matter volume, and cerebrospinal fluid volume were computed using CAT-12.

### 2.1. Data Collection

We collected data from Alzheimer's disease neuroimaging initiative (ADNI) and Australian imaging, biomarker & lifestyle (AIBL) datasets for our model training and testing, respectively. ADNI is a longitudinal multicenter study aimed to explore clinical, imaging, genetic, and biochemical biomarkers for the early detection and tracking of AD (Mueller et al., 2005). AIBL is the largest study of its kind in Australia designed to detect biomarkers, cognitive characteristics, and lifestyle factors that affect the progression of symptomatic AD. The data from ADNI were randomly divided into two groups for training and validation, and AIBL data was used for multi-site testing. The criterion for subject selection included age ≥55, and *T*_1_-weighted MRI scans. Cases were excluded including Alzheimer's disease with mixed dementia, non-Alzheimer's disease dementia, severe depression, stroke, brain tumors, and history of severe traumatic brain injury, as well as incident major systemic illnesses. The inclusion and exclusion criterion was referred to in the baseline recruitment protocol developed by the ADNI study, and the same selection criteria were applied to an other study (Qiu et al., 2020). Finally, 415 NC samples and 351 AD samples were selected from the ADNI dataset, and 284 NC samples and 70 samples were selected from the AIBL dataset. The samples statistics overview is shown in Table 1.

**Table 1:** Participant demographics overview of ADNI and AIBL dataset.

**Dataset**	**Group**	**N**	**Age**	**Gender**
AIBL	AD	70	72.7 ± 7.94[93.0,55.0]	27M/43F
	NC	284	72.4 ± 6.50[92.0,60.0]	128M/156F
ADNI	AD	351	75.6 ± 7.96[91.0,55.0]	192M/159F
	NC	415	76.19 ± 6.33[95.0,56.0]	206M/209F

*Values are presented as mean±std [range]. M, male; F, female*.

### 2.2. Structural MRI Scan Processing

All the structural MRI data in this study were in NIFTI format. We adopted the FSL package (Woolrich et al., 2009) for data processing, where the FSLReorient2STD toolkit was used for redirection, BET toolkit for skull removal, and Flirt toolkit for registration structural MRI to the MNI152 standard brain template [181*217*181]. After structural MRI scans image registration, we normalized the intensities of all the voxels. Then we adjusted the intensity of these voxels and other outliers by clipping them to the range: [-1,2.5], following the method in Qiu et al. (2020). It is well known that Alzheimer's disease leads to significant structural changes in hippocampus-related regions. Wen et al. (2020) chose the hippocampus as ROI and proved a cubic patch [50*50*50] enclosed hippocampus region can be used for training. In our study, we manually cropped a larger [80*100*80] patch to make sure it includes all the hippocampus (left and right).

### 2.3. Morphological Metrics

The adopted morphological metrics derived from ADNI and AIBL in this study included gray matter volume, white matter volume, and cerebrospinal fluid volume. The morphological metrics were extracted using CAT-12. First, the structural MRI scans were manually oriented to place the anterior commissure aligned to the MNI template. Second, the structural MRI data was skull stripped and corrected for bias-field inhomogeneity. Third, the images were segmented into GM, WM, and CSF using a unified segmentation method, and then normalized into the MNI space with the DARTEL algorithm. Specially, an integrated modulation step was applied to preserve volume information at each tissue voxel. Finally, the segmented scans were smoothed with an isotropic 4 mm full width half maximum (FWHM) Gaussian kernel. After that, the GM, WM, and CSF volumes were extracted from the smoothed images using hammers atlas which are available within the CAT-12.

### 2.4. Re-CNN

**Figure 1:**
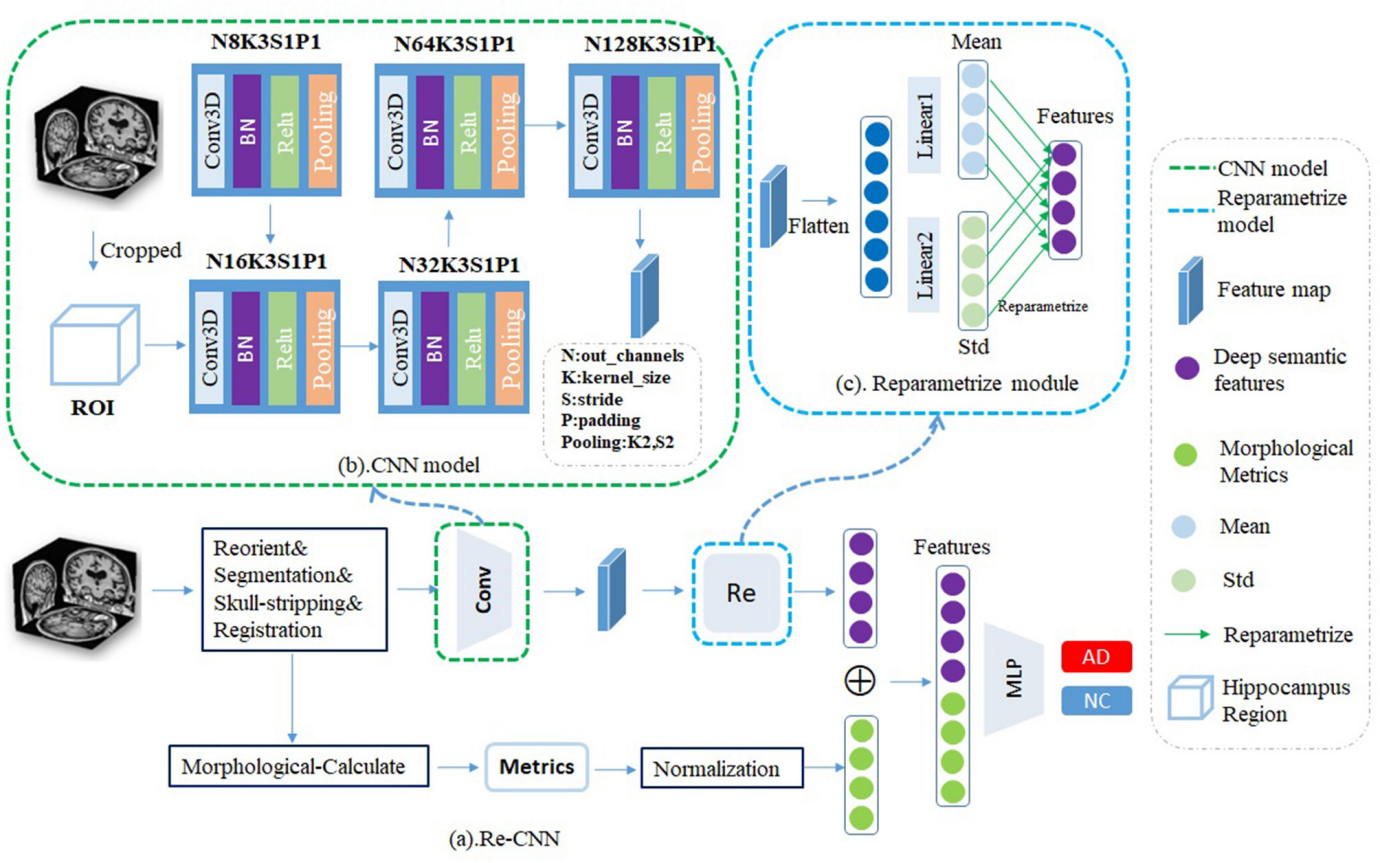
**(a)** Our reparametrized convolutional neural network (Re-CNN) consisted with two components, **(b)** CNN model, and **(c)** Reparametrized module.

Our Re-CNN model consists of the CNN model and reparametrized module (as shown in Figure 1). CNN model inputs the cropped hippocampus patches and generates the original deep semantic features, reparametrized module constrains the similarity of the original deep semantic features and generates the most distinguishing features. Finally, we concatenate the deep semantic features and the morphological metrics together as inputs to a multi-layer perceptron classifier for AD recognition.

The CNN model includes four convolution modules, each module containing Conv3D, BatchNorm3D, ReLU, and MaxPooling3D. The Conv3D is used for local feature extraction, BatchNorm3D is used for feature normalization, and the ReLU is used to add nonlinearity. To capture global semantic features, we use the PadMaxPool3d layer (Wen et al., 2020) to reduce the dimension of the input, so the pixels on the feature map have a larger receptive field in the original patches. The CNN model is shown in Figure 1B. After passing *k* = 5 convolution modules, the output size of patches is [128,3,4,3], which means that we have obtained 128 feature maps with the size of [3,4,3]. The corresponding size of the layer k is calculated as follows:


(1) $$\begin{array}{l} l_{k}=l_{k-1}+\left[\left(f_{k}-1\right)*\overset{i=1}{\underset{k-1}{\prod }}s_{i}\right] \end{array}$$

Where *l*_*k*−1_ represents the size of the receptive field corresponding to layer *k*−1, *f*_*k*_ represents the kernel size of layer *k*, *S*_*i*_ represents the stride. So each pixel in the feature map can correspond to the 32*32*32 region on the original map. A similar size was also used by Wen et al. (2020), and they proved that the size of the receptive field can effectively recognize AD from NC.

The reparametrized module was designed to obtain the most differentiating features. First, we flatten the features (size from [128,3,4,3] to [128*3*4*3]) and send them into two different fully connected layers *f*_*m*_ and *f*_*s*_. The outputs of *f*_*m*_ and *f*_*s*_ can be viewed as mean value α and variance σ of the distribution of the features, respectively, then the features were randomly sampled according to α and σ. Assuming there are distributions in the hidden space, the reparametrized process can be expressed by the following formula:


(2) $$\begin{array}{l} Z=\varepsilon *\sigma +\mu \end{array}$$

Where *Z* represents the features after the reparametrized process, ε represents the *n* values created by standard normal distribution ε~*N*(0, 1), SD σ can be formulated as:


(3) $$\begin{array}{l} \sigma =e^{f_{s}\left(h\right)/2} \end{array}$$

Where *h* represents initial features, *f*_*s*_(*h*) represents linear transformation by fully connected layer *f*_*s*_, Mean value μ can be formulated as:


(4) $$\begin{array}{l} \mu =f_{m}\left(h\right) \end{array}$$

*f*_*m*_(*h*) represents linear transformation by a fully connected layer. According to Equation 2, it is obvious that the processed features can be derived linearly from the normal distribution ε~*N*(0, 1). We can achieve dimension reduction by modifying the number of neurons in the full connection layers *f*_*m*_ and *f*_*s*_. The proposed reparametrized module can predict features distribution instead of obtaining features directly, our reparametrized function can increase the diversity of the features.

### 2.5. Loss Function

**Figure 2:**
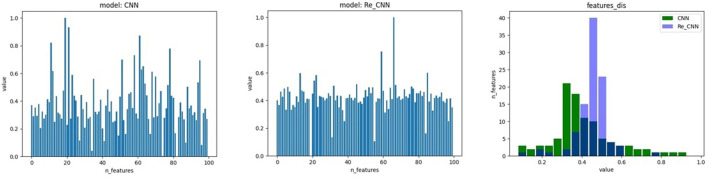
The feature distribution of hidden space, 1) the feature distribution of conventional CNN, 2) the feature distribution of Re-CNN, 3) the feature distribution histogram of the two models.

In this study, we proposed a loss function based on cross entropy and KL divergence. The cross entropy loss *l*_*ce*_ is used to increase the difference in the features. The feature distribution in hidden space became discontinuous due to the cross entropy (as shown in Figure 2A). Therefore, KL divergence loss *l*_*kl*_ was used to restrict feature distribution to standard normal distribution. Our loss function can be expressed as:


(5) $$\begin{array}{l} l=l_{ce}+\alpha l_{kl} \end{array}$$

α is an adjustable hyper-parameter representing the balance proportionality between the two loss functions, KL divergence loss function lets the feature distribution in hidden space become more continuous (as shown in Figure 2B). The cross entropy loss function *l*_*ce*_ can be formulated as:


(6) $$\begin{array}{l} l_{ce}=\frac{1}{N}\underset{i}{\sum }L_{i}=-\frac{1}{N}\underset{i}{\sum }\overset{M}{\underset{c}{\sum }}y_{ic}log\left(p_{ic}\right) \end{array}$$

Where *y*_*ic*_ is set to 1 if the prediction category is equal to the label category, otherwise is set to 0. *p*_*ic*_ is the probability that sample *i* belongs to class *c*. KL divergence loss *l*_*kl*_ can be formulated as:


(7) $$\begin{array}{l} l_{kl}=KL\left(p\left(x\right),q\left(x\right)\right)=\sum p\left(x\right)log\frac{p\left(x\right)}{q\left(x\right)} \end{array}$$

Where *p*(*x*) is a standard normal distribution *N*(0, 1), *q*(*x*) is features distribution of hidden space. All feature distributions in hidden space are constrained to *N*(0, 1) through the KL divergence loss function. The KL divergence loss can be simplified by μ and σ of the hidden space feature distribution:


(8) $$\begin{array}{l} l_{kl}=\frac{1}{2}\overset{J}{\underset{j=1}{\sum }}\left[\left(\mu_{j}^{2}+\sigma_{j}^{2}\right)-1-log\sigma_{j}^{2}\right] \end{array}$$

Where μ and σ in Equations 3 and 4 have the same meaning, *J* denotes the feature dimension of hidden space and *j* denotes the *j*_*th*_ feature. Our loss function ensures that the features' similarity is maximized while the differential features are retained.

### 2.6. Experiments

A total of 415 NC samples and 351 AD samples were selected from ADNI dataset for the experiments, we applied 80% of samples for model training, and 20% of samples for model validation. We fixed random seeds to make sure the same samples were utilized in the training and validation of all models. We modified the seed value to implement random cross-validation. As for the sample imbalance of AD and NC groups in the training set, we adopted oversampling method to give more weight to the AD group. AIBL was used to test directly as external multi-site data.

Our Re-CNN was implemented using Python with the Pytorch package, and the computer contains a single GPU (NVIDIA RTX 3090 24GB). We adopted random initialization weight to train Re-CNN from scratch. The Adam optimizer with a 0.0005 learning rate and a mini-batch size of 32 was set for training. The Re-CNN was trained for 120 epochs, which took around 7 min (i.e., 3.54 s for each epoch). The initial learning rate was set to 0, the learning rate warm-up method was used in the first two epochs and the rate increased according to the amount of training data. Finally, the learning rate remained unchanged at 0.0005 after the second epoch. Then, we adjusted the learning rate dynamically according to the number of epochs (learning rate shrinks 5 times per 40 epochs) to achieve better convergence.

### 2.7. Performance Metrics

The AD recognition performance was evaluated by four metrics, including accuracy (ACC), sensitivity (SEN), specificity (SPE), and area under the receiver operating characteristic curve (AUC). These metrics are defined as $ACC=\frac{TP+TN}{TP+TN+FP+FN}$, $SE=\frac{TP}{TP+FN}$, $SP=\frac{TN}{TN+FP}$, where TP, TN, FP, and FN denote, the true positive, true negative, false positive, and false negative values, respectively.

## 3. Results

**Figure 3:**
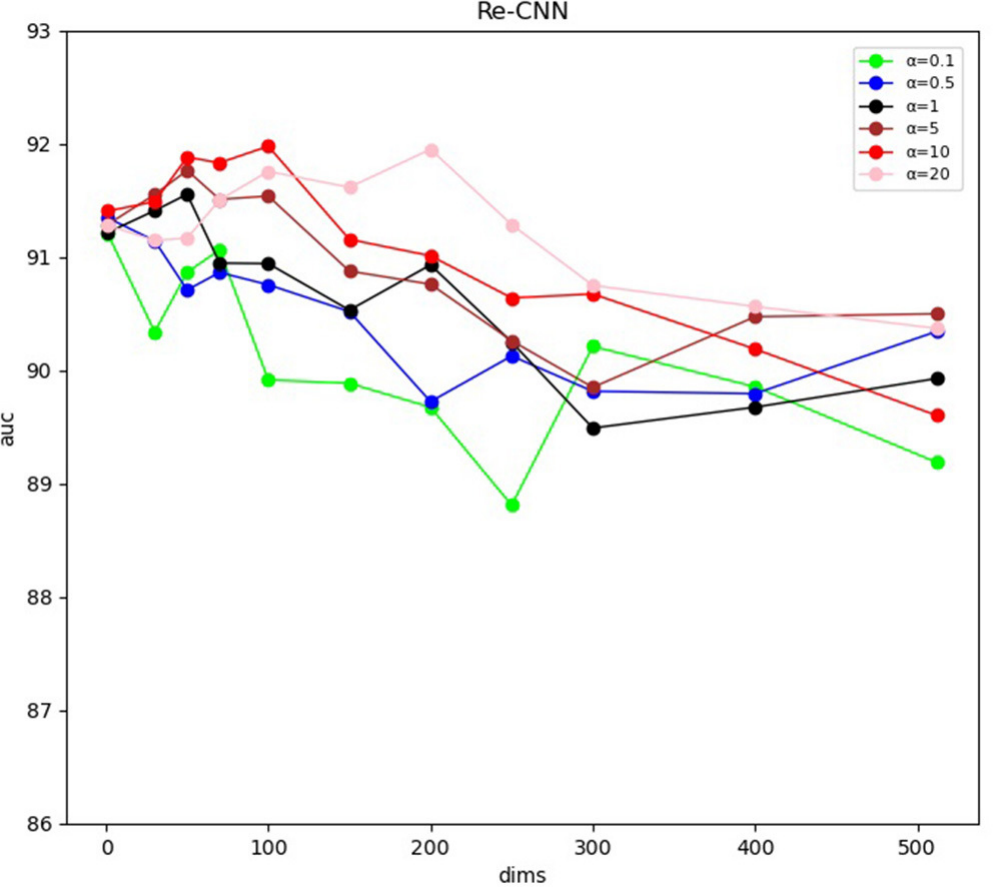
Area under the receiver operating characteristic curve at different semantic features dimension and hyperparameter α.

Our Re-CNN model can differentiate AD from NC by applying morphological metrics and most distinguishing deep semantic features. The experiments results were shown in (Figure 3 and Table 1). Table 1 shows the AUC of the model at different semantic features dimension and hyperparameter α, all the results were validated five times to reduce randomness, and the mean and SD of AUC were illustrated. Figure 3 visualized the AUC at different semantic features dimension and hyperparameter α. Our model achieved the optimal AUC when the hyperparameter α is 10, and the feature dimension is 100.

**Figure 4:**
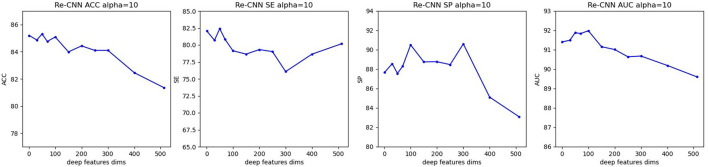
Precision, sensitivity, specificity, and AUC of our model (when α equals 10, semantic features dimension increases from 0 to 512). **(A)** Re-CNN. **(B)** CNN model. **(C)** Reparametrize module.

When hyperparameter α is equal to 10, at different semantic features dimension, precision, sensitivity, specificity, and AUC of our model were shown in Figure 4.

We compared our model with conventional CNN, MLP, SVM, random forest, and Xgboost, the descriptive information about these five models was added in the Supplementary Materials. All the models used the same training and validation data as our Re-CNN, as shown in Section 2.6. All the results in Tables 2, 3 were validated five times (random cross-validation based on different seeds), and the mean and SD of ACC, SP, SE, and AUC of all the models are shown in Tables 2, 3.

**Table 2:** Area under the receiver operating characteristic curve (AUC) of our Re-CNN model at different semantic features dimension and hyperparameter α.

**Features**	**α = 0.1**	**α = 0.5**	**α = 1**	**α = 5**	**α = 10**	**α = 20**
mf+0	0.8977 ± 0.020	0.8999 ± 0.015	0.9033 ± 0.015	0.8950 ± 0.012	0.9050 ± 0.016	0.9020 ± 0.020
mf+1	0.9121 ± 0.019	0.9135 ± 0.015	0.9122 ± 0.012	0.9129 ± 0.011	0.9140 ± 0.013	0.9128 ± 0.026
mf+30	0.9034 ± 0.016	0.9114 ± 0.014	0.9141 ± 0.012	0.9155 ± 0.020	0.9149 ± 0.024	0.9114 ± 0.025
mf+50	0.9086 ± 0.019	0.9071 ± 0.013	0.9155 ± 0.015	0.9176 ± 0.018	0.9188 ± 0.022	0.9117 ± 0.026
mf+70	0.9106 ± 0.014	0.9086 ± 0.014	0.9095 ± 0.019	0.9151 ± 0.022	0.9183 ± 0.025	0.9150 ± 0.024
mf+100	0.8991 ± 0.015	0.9075 ± 0.019	0.9094 ± 0.017	0.9154 ± 0.024	**0.9198** **±0.023**	0.9175 ± 0.027
mf+150	0.8988 ± 0.012	0.9052 ± 0.019	0.9053 ± 0.026	0.9087 ± 0.021	0.9115 ± 0.029	0.9161 ± 0.022
mf+200	0.8967 ± 0.011	0.8972 ± 0.020	0.9093 ± 0.025	0.9076 ± 0.021	0.9101 ± 0.019	0.9195 ± 0.027
mf+250	0.8881 ± 0.012	0.9013 ± 0.020	0.9025 ± 0.021	0.9026 ± 0.025	0.9064 ± 0.034	0.9128 ± 0.015
mf+300	0.9021 ± 0.013	0.8981 ± 0.018	0.8949 ± 0.020	0.8985 ± 0.024	0.9067 ± 0.026	0.9075 ± 0.019
mf+400	0.8985 ± 0.015	0.8979 ± 0.024	0.8967 ± 0.020	0.9047 ± 0.020	0.9019 ± 0.031	0.9056 ± 0.024
mf+512	0.8919 ± 0.013	0.9034 ± 0.025	0.8993 ± 0.021	0.9050 ± 0.027	0.8960 ± 0.020	0.9037 ± 0.017

*The mf in this table represents morphological metrics.*

*Bold value indicates Re-CNN achieves best AUC when α equals to 10 and semantic features dimension equals to 100*.

**Table 3:** ADNI random cross-validation, ACC, SE, SP, and AUC of all the models.

**Models**	**CNN**	**Random Forest**	**SVM**	**Xgboost**	**MLP**	**Re-CNN**
ACC	0.8201 ± 0.018	0.8398 ± 0.018	0.7954 ± 0.021	0.8549 ± 0.026	0.8062 ± 0.030	0.8508 ± 0.027
SE	0.8652 ± 0.035	0.7949 ± 0.038	0.7480 ± 0.044	0.8168 ± 0.040	0.7870 ± 0.089	0.7914 ± 0.047
SP	0.7639 ± 0.031	0.8807 ± 0.015	0.8386 ± 0.030	0.8900 ± 0.024	0.8372 ± 0.086	**0.9050** ±**0.020**
AUC	0.8938 ± 0.028	0.9145 ± 0.014	0.8600 ± 0.018	0.9275 ± 0.014	0.9020 ± 0.020	0.9198 ± 0.052

*Bold value indicates Re-CNN achieves best SP*.

**Figure 5:**
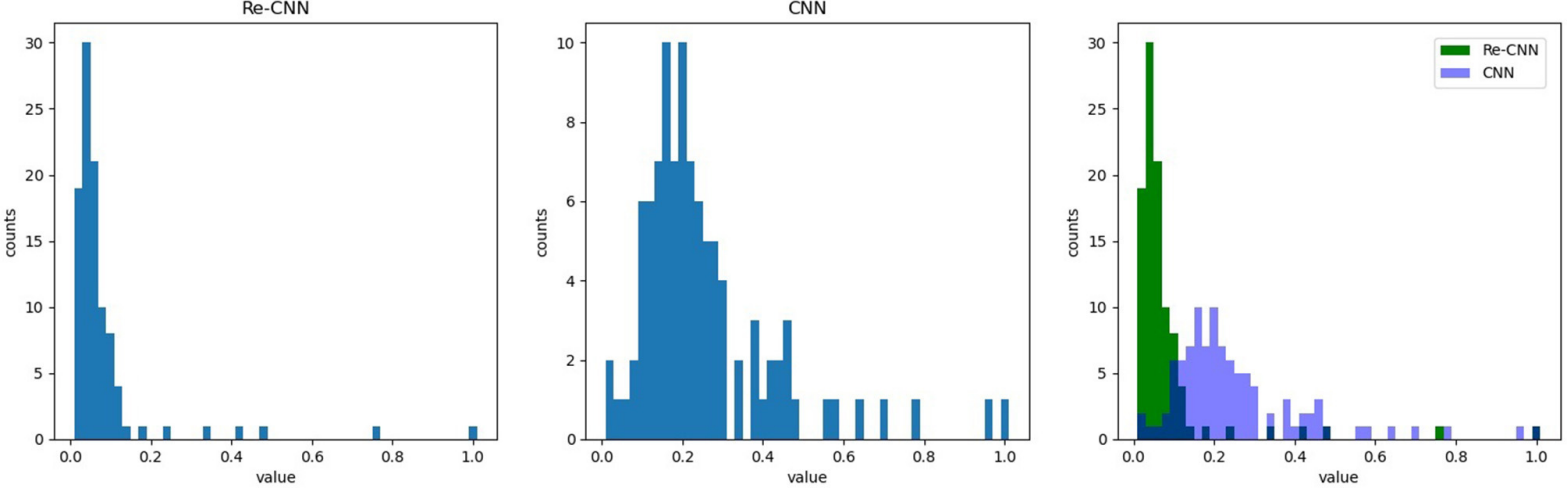
Re-CNNs' α equals 1, semantic features dimension equals 100, neuron weights corresponding to the semantic features are shown in the left figure. The neuron weights of the first layer of MLP. The first 100 neuron weights from CNN are shown in the middle figure. The right figure shows the neuron weights histogram of the two models.

In addition, we compared the weights of the neurons in MLP corresponding to deep semantic features in conventional CNN and our Re-CNN, results showed that conventional CNN had much more high neurons weights than our Re-CNN, as shown in Figure 5. The left figure shows the first 100 neuron weights corresponding to conventional CNN. The middle figure shows the weights for Re-CNN when alpha equals 1 and the deep semantic feature dimension equals 100. The right figure shows the neuron weights histogram of the two models.

## 4. Discussion

To improve the accuracy of discriminating AD from NC by structural MRI, we built an end-to-end model Re-CNN that integrates morphological metrics and deep semantic features from structural MRI for AD recognition. To address the problem of redundancy and correlation of the deep semantic features, we designed a reparametrized module to impose similarity constraints on features and retain the most distinguishing features.

We constructed a new loss function by integrating cross entropy and KL divergence loss functions. The weight coefficient α of KL divergence is an adjustable hyper-parameter to balance proportionality between the two loss functions. When α was set to small values, i.e., weak similarity constraint, a large number of redundancy features were extracted by the CNN model. The recognition performance of our model [The AUC is about 0.90, as shown in Figure 3 (α = 0.1)] cannot be further improved since the weak similarity constraint. As the α increased, the redundant features were filtered out and the most distinguishing features were extracted through the model. It should be noted that the performance of our Re-CNN cannot be improved consistently with the increase of hyperparameter α, as shown in Figure 3 (α = 5, 10, 20), when the α was very large, the deep semantic features are over-constrained, most of the deep semantic feature distributions were restricted to the standard normal distribution, the distinguishing ability of deep semantic features was gradually lost. When α was set to 10, our Re-CNN model achieved the best performance (AUC=0.9198 ± 0.020).

In our study, the deep semantic features dimension also affects the performance of our Re-CNN model. When the number of deep semantic features was set to 0 or 1, there is no deep semantic features adopted and the Re-CNN model degenerated to MLP (The AUC is about 0.90, as shown in Table 2, mf+0,1). As the deep semantic features increased, recognition performance declined when α was set to 0.1 or 0.5 (as shown in Figure 3 blue and green line) since the small α values can not constrain a large number of deep semantic features. However, the performance increased when α was set to 5,10,20 (as shown in Figure 3 in brown, red, and pink lines) since the appropriate dimension of deep semantic features were constrained and most distinguishing features were extracted. When the number of deep semantic features was set to 100 and α was set to 10, our Re-CNN model achieved the best result.

In addition, the MLP module would respond to different features with different weights. It could be clearly seen that many neuron weights in the Re-CNN almost converge to 0, while the neuron weights in the conventional CNN were more scattered (as shown in Figure 5). The reason was that high differentiation features would correspond to larger weights, and redundant features corresponding weights would tend to 0. Redundant features played little role in recognition tasks, so the weights of the neurons connected converge to 0. The conventional CNN did not impose similarity constraints on the features, a large number of redundant features were fed into the classifier, resulting in low recognition accuracy.

Most of the deep learning based AD recognition models achieved relatively high recognition accuracy on structural MRI. It is difficult to compare the performance of various models because different models used different data sets. On the same structural MRI data set, we compared the recognition performance of our Re-CNN model with conventional CNN, our model imposed similarity constraints on features and retained the most distinguishing features. For the ADNI random cross-validation (as shown in Table 3), the ACC, SP, and AUC of our Re-CNN model (under α = 10, and the deep semantic feature dimension is 100) outperformed the conventional CNN model. For the AIBL multi-site testing (as shown in Table 4), the SE and AUC of our Re-CNN model were superior to the conventional CNN model. The results showed that deep semantic features have limitations on improving AD recognition performance, but constrained deep semantic features with the reparametrized module can improve recognition performance.

**Table 4:** AIBL multi-site testing, ACC, SE, SP, and AUC of all the models.

**Models**	**CNN**	**Random Forest**	**SVM**	**Xgboost**	**MLP**	**Re-CNN**
ACC	0.8699 ± 0.016	0.8575 ± 0.005	0.8481 ± 0.005	0.8584 ± 0.016	0.8564 ± 0.008	0.8541 ± 0.008
SE	0.4744 ± 0.034	0.5833 ± 0.127	0.4857 ± 0.128	0.5976 ± 0.095	0.5380 ± 0.073	0.5074 ± 0.068
SP	0.9481 ± 0.019	0.9084 ± 0.007	0.9178 ± 0.007	0.9097 ± 0.022	0.9213 ± 0.018	0.9356 ± 0.022
AUC	0.7797 ± 0.005	0.7999 ± 0.059	0.7494 ± 0.073	0.8012 ± 0.068	0.7846 ± 0.064	**0.8034** **±0.008**

*Bold value indicates Re-CNN achieves best AUC*.

We compared the Re-CNN model with SVM, random forest, MLP, and Xgboost on morphological metrics extract from the same structural MRI data set (as shown in Table 3,4). Compared to SVM, our Re-CNN has better ACC, SE, SP, and AUC in both ADNI and AIBL. These results showed the effectiveness of our Re-CNN. Compared to MLP, Re-CNN utilized the same classifier structure as MLP. Our model combined deep semantic features with morphological metrics and achieved the best ACC, SP, SE, and AUC on ADNI and the best SP and AUC on AIBL. The results demonstrated that recognition performance can be improved by applying constrained deep semantic features with the same classifier (MLP). Compared with some stronger classifiers, such as random forest and Xgboost, our model adopted a relatively weak classifier MLP and reached comparable results (the best SP on ADNI and the best AUC on AIBL) with random forest and Xgboost, which means the extracted features with Re-CNN are effective.

## 5. Conclusion

We proposed an end-to-end neural network model Re-CNN, applying morphological metrics and deep semantic features from structural MRI to distinguish AD from NC. The reparametrized module imposed similarity constraints on deep semantic features and retained the most distinguishing features. Our model worked with relatively high recognition and generalization performance and was superior to the conventional CNN model.

## Data Availability Statement

The original contributions presented in the study are included in the article/Supplementary Material, further inquiries can be directed to the corresponding author. The datasets analyzed for this study can be found in the ADNI [http://adni.loni.usc.edu/] and AIBL [https://aibl.csiro.au/].

## Author Contributions

ZC: methodology, architecture design, and writing the original manuscript. XM and RC: data collection and preprocessing. PF: programming. HL: conceptualization and manuscript revision. All the authors read and approved the final manuscript.

## Funding

This study was supported by Beijing Natural Science Foundation no. L192044.

## Conflict of Interest

The authors declare that the research was conducted in the absence of any commercial or financial relationships that could be construed as a potential conflict of interest.

## Publisher's Note

All claims expressed in this article are solely those of the authors and do not necessarily represent those of their affiliated organizations, or those of the publisher, the editors and the reviewers. Any product that may be evaluated in this article, or claim that may be made by its manufacturer, is not guaranteed or endorsed by the publisher.

## References

[B1] Aderghal K. Khvostikov A. Krylov A. Benois-Pineau J. Afdel K. Catheline G. (2018). “Classification of alzheimer disease on imaging modalities with deep cnns using cross-modal transfer learning,” in 2018 IEEE 31st International Symposium on Computer-Based Medical Systems (CBMS) (Karlstad: IEEE), 345–350.

[B2] He K. Zhang X. Ren S. Sun J. (2016). “Deep residual learning for image recognition,” in Proceedings of the IEEE Conference on Computer Vision and Pattern Recognition (Las Vegas, NV: IEEE), 770–778.

[B3] Huang G. Liu Z. Van Der Maaten L. Weinberger K. Q. (2017). “Densely connected convolutional networks,” in Proceedings of the IEEE Conference on Computer Vision and Pattern Recognition (Honolulu, HI: IEEE), 4700–4708.

[B4] Jagust W.. (2013). Vulnerable neural systems and the borderland of brain aging and neurodegeneration. Neuron 77, 219–234. 10.1016/j.neuron.2013.01.002 23352159 PMC3558930

[B5] Jing B. Liu B. Li H. Lei J. Wang Z. Yang Y.. (2018). Within-subject test-retest reliability of the atlas-based cortical volume measurement in the rat brain: a voxel-based morphometry study. J. Neurosci. Methods 307:46–52. 10.1016/j.jneumeth.2018.06.022 29960027 PMC6461491

[B6] Koikkalainen J. Lötjönen J. Thurfjell L. Rueckert D. Waldemar G. Soininen H.. (2011). Multi-template tensor-based morphometry: application to analysis of Alzheimer's disease. Neuroimage 56, 1134–1144. 10.1016/j.neuroimage.2011.03.029 21419228 PMC3554792

[B7] Lian C. Liu M. Zhang J. Shen D. (2018). Hierarchical fully convolutional network for joint atrophy localization and alzheimer's disease diagnosis using structural mri. IEEE Trans. Pattern. Anal. Mach. Intell. 42, 880–893. 10.1109/TPAMI.2018.2889096 30582529 PMC6588512

[B8] Long Z. Huang J. Li B. Li Z. Li Z. Chen H.. (2018). A comparative atlas-based recognition of mild cognitive impairment with voxel-based morphometry. Front. Neurosci. 12:916. 10.3389/fnins.2018.00916 30574064 PMC6291519

[B9] Ma Z. Jing B. Li Y. Yan H. Li Z. Ma X.. (2020). Identifying mild cognitive impairment with random forest by integrating multiple mri morphological metrics. J. Alzheimers Dis. 73, 991–1002. 10.3233/JAD-190715 31884464

[B10] Mueller S. G. Weiner M. W. Thal L. J. Petersen R. C. Jack C. Jagust W.. (2005). The Alzheimer's disease neuroimaging initiative. Neuroimaging Clin. North Am. 15, 869. 10.1016/j.nic.2005.09.008 PMC2376747 16443497

[B11] Pan D. Zeng A. Jia L. Huang Y. Frizzell T. Song X. (2020). Early detection of alzheimer's disease using magnetic resonance imaging: a novel approach combining convolutional neural networks and ensemble learning. Frontiers in neuroscience 14:259. 10.3389/fnins.2020.00259 32477040 PMC7238823

[B12] Park H. Yang J.-J. Seo J. Lee J.-m. (2012). Dimensionality reduced cortical features and their use in the classification of Alzheimer's disease and mild cognitive impairment. Neurosci. Lett. 529, 123–127. 10.1016/j.neulet.2012.09.011 23000551

[B13] Pennanen C. Kivipelto M. Tuomainen S. Hartikainen P. Hänninen T. Laakso M. P.. (2004). Hippocampus and entorhinal cortex in mild cognitive impairment and early ad. Neurobiol. Aging 25, 303–310. 10.1016/S0197-4580(03)00084-8 15123335

[B14] Qiu S. Joshi P. S. Miller M. I. Xue C. Zhou X. Karjadi C.. (2020). Development and validation of an interpretable deep learning framework for Alzheimer's disease classification. Brain 143, 1920–1933. 10.1093/brain/awaa137 32357201 PMC7296847

[B15] Schmitter D. Roche A. Maréchal B. Ribes D. Abdulkadir A. Bach-Cuadra M.. (2015). An evaluation of volume-based morphometry for prediction of mild cognitive impairment and Alzheimer's disease. Neuroimage Clin. 7, 7–17. 10.1016/j.nicl.2014.11.001 25429357 PMC4238047

[B16] Tan M. Le Q. (2019). “Efficientnet: rethinking model scaling for convolutional neural networks,” in International Conference on Machine Learning (PMLR), (Mingxing Tan; Quoc V. Le) 6105–6114.

[B17] Wen J. Thibeau-Sutre E. Diaz-Melo M. Samper-González J. Routier A. Bottani S.. (2020). Convolutional neural networks for classification of alzheimer's disease: overview and reproducible evaluation. Med. Image Anal. 63, 101694. 10.1016/j.media.2020.101694 32417716

[B18] Woolrich M. W. Jbabdi S. Patenaude B. Chappell M. Makni S. Behrens T.. (2009). Bayesian analysis of neuroimaging data in fsl. Neuroimage 45, S173–S186. 10.1016/j.neuroimage.2008.10.055 19059349

[B19] Zhao J. Ma Z. Chen F. Li L. Ren M. Li A.. (2021). Human immune deficiency virus-related structural alterations in the brain are dependent on age. Hum. Brain Mapp. 42, 3131–3140. 10.1002/hbm.25423 33755269 PMC8193536

