# Supplementary material for: A Reparametrized CNN Model to Distinguish Alzheimer's Disease Applying Multiple Morphological Metrics and Deep Semantic Features From Structural MRI
Source: Front Aging Neurosci. 2022 May 26;14:856391. doi: 10.3389/fnagi.2022.856391 (PMC9204294; doi:10.3389/fnagi.2022.856391)
Supplement: Supplementary file 1 [file Data_Sheet_1.pdf]

Supplementary Material

1 SUPPLEMENTARY TABLES

Table S1. Re-CNN framework (dims=100).

Layer	Specification	Size of output
Input		(1,80,100,80)
3D Conv	in_c=1,out_c=8,kernel_size=3,stride=1,padding=1	(8,80,100,80)
BN	eps=1e-5,momentum=0.1,affine=True	(8,80,100,80)
Relu	inplace=False	(8,80,100,80)
PadMaxPool3d	kernel_size=2,stride=2	(8,40,50,40)
3D Conv	in_c=8,out_c=16,kernel_size=3,stride=1,padding=1	(16,40,50,40)
BN	eps=1e-5,momentum=0.1,affine=True	(16,40,50,40)
Relu	inplace=False	(16,40,50,40)
PadMaxPool3d	kernel_size=2,stride=2	(16,20,25,20)
3D Conv	in_c=16,out_c=32,kernel_size=3,stride=1,padding=1	(32,20,25,20)
BN	eps=1e-5,momentum=0.1,affine=True	(32,20,25,20)
Relu	inplace=False	(32,20,25,20)
PadMaxPool3d	kernel_size=2,stride=2	(32,10,13,10)
3D Conv	in_c=32,out_c=64,kernel_size=3,stride=1,padding=1	(64,10,13,10)
BN	eps=1e-5,momentum=0.1,affine=True	(64,10,13,10)
Relu	inplace=False	(64,10,13,10)
PadMaxPool3d	kernel_size=2,stride=2	(64,5,7,5)
3D Conv	in_c=64,out_c=128,kernel_size=3,stride=1,padding=1	(128,5,7,5)
BN	eps=1e-5,momentum=0.1,affine=True	(128,5,7,5)
Relu	inplace=False	(128,5,7,5)
PadMaxPool3d	kernel_size=2,stride=2	(128,3,4,3)
flatten		(4608)
f_m	in_c=4608,out_c=100,bias=True	(100)
f_s	in_c=4608,out_c=100,bias=True	(100)
Linear	in_c=304,out_c=1024,bias=True	(1024)
Relu	inplace=False	(1024)
Linear	in_c=1024,out_c=50,bias=True	(50)
Relu	inplace=False	(50)
Linear	in_c=50,out_c=2,bias=True	(2)

Table S2. conventional CNN framework.

Layer	Specification	Size of output
Input		(1,80,100,80)
3D Conv	in_c=1,out_c=8,kernel_size=3,stride=1,padding=1	(8,80,100,80)
BN	eps=1e-5,momentum=0.1,affine=True	(8,80,100,80)
Relu	inplace=False	(8,80,100,80)
PadMaxPool3d	kernel_size=2,stride=2	(8,40,50,40)
3D Conv	in_c=8,out_c=16,kernel_size=3,stride=1,padding=1	(16,40,50,40)
BN	eps=1e-5,momentum=0.1,affine=True	(16,40,50,40)
Relu	inplace=False	(16,40,50,40)
PadMaxPool3d	kernel_size=2,stride=2	(16,20,25,20)
3D Conv	in_c=16,out_c=32,kernel_size=3,stride=1,padding=1	(32,20,25,20)
BN	eps=1e-5,momentum=0.1,affine=True	(32,20,25,20)
Relu	inplace=False	(32,20,25,20)
PadMaxPool3d	kernel_size=2,stride=2	(32,10,13,10)
3D Conv	in_c=32,out_c=64,kernel_size=3,stride=1,padding=1	(64,10,13,10)
BN	eps=1e-5,momentum=0.1,affine=True	(64,10,13,10)
Relu	inplace=False	(64,10,13,10)
PadMaxPool3d	kernel_size=2,stride=2	(64,5,7,5)
3D Conv	in_c=64,out_c=128,kernel_size=3,stride=1,padding=1	(128,5,7,5)
BN	eps=1e-5,momentum=0.1,affine=True	(128,5,7,5)
Relu	inplace=False	(128,5,7,5)
PadMaxPool3d	kernel_size=2,stride=2	(128,3,4,3)
flatten		(4608)
Linear	in_c=4608,out_c=1024,bias=True	(1024)
Relu	inplace=False	(1024)
Linear	in_c=1024,out_c=50,bias=True	(50)
Relu	inplace=False	(50)
Linear	in_c=50,out_c=2,bias=True	(2)

Table S3. The hyperparameter setting of SVM, random forest and xgboost.

model	Specification
random_forest	n_estimators=25,random_state=0,bootstrap=False
SVM	kernal='poly',probability=True
xgboost	n_estimators=100,max_depth=6

Table S4. MLP framework.

Layer	Specification	Size of output
Linear	in_c=204,out_c=1024,bias=True	(1024)
Relu	inplace=False	(1024)
Linear	in_c=1024,out_c=50,bias=True	(50)
Relu	inplace=False	(50)
Linear	in_c=50,out_c=2,bias=True	(2)
